# Safety and tolerability of etirinotecan pegol in advanced breast cancer: analysis of the randomized, phase 3 BEACON trial

**DOI:** 10.1186/s40064-016-2446-4

**Published:** 2016-07-08

**Authors:** Javier Cortés, Hope S. Rugo, Chris Twelves, Ahmad Awada, Edith A. Perez, Seock-Ah Im, Carol Zhao, Ute Hoch, Denise Tomkinson, James Buchanan, Mary Tagliaferri, Alison Hannah, Joyce O’Shaughnessy

**Affiliations:** Ramon y Cajal University Hospital, Madrid, Spain; Vall d’Hebron Institute of Oncology (VHIO), Vall d’Hebron 119-129, Barcelona, Spain; University of California, San Francisco, CA USA; St. James’s University Hospital, University of Leeds, Leeds, UK; Medical Oncology Clinic, Jules Bordet Institute, Brussels, Belgium; Mayo Clinic, Jacksonville, FL USA; Cancer Research Institute, Seoul National University Hospital, Seoul National University College of Medicine, Seoul, Korea; Nektar Therapeutics, San Francisco, CA USA; Consultant, Sebastopol, CA USA; Baylor-Sammons Cancer Center, Texas Oncology, U.S. Oncology, Dallas, TX USA

**Keywords:** Etirinotecan pegol, Metastatic breast cancer, Chemotherapy safety, Chemotherapy side effects

## Abstract

**Purpose:**

New treatments with novel mechanisms of action and non-overlapping toxicities are needed for patients with metastatic breast cancer. Etirinotecan pegol (EP) is a long-acting topoisomerase-I inhibitor with a unique toxicity profile. The randomized phase 3 BEACON study that compared EP to treatment of physician’s choice (TPC) demonstrated its clinical activity. We now present detailed safety data from the BEACON trial.

**Methods:**

Patients with locally recurrent or metastatic breast cancer who had received at least two prior cytotoxic regimens for advanced disease were randomized to EP or TPC. Prior treatment with an anthracycline, a taxane and capecitabine was required. The frequencies of treatment-emergent AEs (TEAEs) and serious TEAEs were evaluated for the safety population, comprising all patients who received at least one dose of assigned treatment.

**Results:**

A total of 831 patients were evaluated (n = 425, EP; n = 406, TPC). Compared with TPC, EP was associated with a slightly higher median relative dose intensity (98.3 vs. 92.8 %, respectively) and significantly fewer grade ≥3 toxicities (48.0 vs. 63.1 %, *P* < 0.0001). The most commonly reported grade ≥3 toxicities in the EP arm were diarrhea (9.6 %) and neutropenia (9.6 %) and in the TPC arm, neutropenia (30.8 %). Median time to onset of grade ≥3 diarrhea was delayed with EP relative to TPC (43 vs. 7 days, respectively).

**Conclusions:**

The differentiated mechanism of action of EP resulted in a safety profile that is substantially distinguished from that of current widely used therapies for the treatment of women with advanced breast cancer.

## Background

Chemotherapy prolongs survival and can improve quality of life (QOL) for patients with metastatic breast cancer (MBC) (National Comprehensive Cancer Network 2015). The use of multiple lines of therapy is limited by emergence of resistance and development of cumulative toxicities. As recognized by the American Society of Clinical Oncology, tolerability is a critical determinant of the clinical value of new cancer drugs (Ellis et al. 2014). For this reason, there is a growing need to more closely scrutinize the safety profiles of drugs in late stage clinical development, particularly when improvements in efficacy are relatively modest.

Etirinotecan pegol (EP) is a novel, long-acting topoisomerase-1 inhibitor designed to improve the pharmacokinetics and tolerability of the prodrug irinotecan. Etirinotecan pegol contains a large-chain polyethylene glycol (PEG) core and four irinotecan molecules that are attached via a cleavable ester-based linker (Hoch et al. 2014). Pegylation facilitates accumulation of the molecule in tumor tissue through the enhanced permeation and retention (EPR) effect, demonstrated in an animal model by high and sustained tumor exposure to SN38 after EP administration (Hoch et al. 2014). Slow hydrolysis of the linker also alters the pharmacokinetics of irinotecan, resulting in prolonged exposure to its active metabolite, SN38. The mean half-life of SN38 was extended from 2 days with conventional irinotecan to 50 days with EP in a phase 1 trial (Von Hoff et al. 2008; Jameson et al. 2013). Early clinical studies showed EP to be active and generally well tolerated in patients with advanced solid tumors, including MBC (Von Hoff et al. 2008; Jameson et al. 2013; Awada et al. 2013). A randomized phase 2 study that assessed two schedules of EP (145 mg/m^2^ every 14 or 21 days) in patients with previously treated MBC demonstrated considerable antitumor activity (objective response rate, 29 %, including two complete responses) (Awada et al. 2013). The every 3-week schedule was selected for further clinical development based on the clinical data, pharmacokinetics, and tolerability demonstrated in this trial.

The randomized phase 3 BEACON (BrEAst Cancer Outcomes with NKTR-102; registered with ClinicalTrials.gov number NCT01492101) study was designed to compare the overall survival (OS) of patients with heavily pretreated, locally recurrent, or metastatic breast cancer treated with EP given every 3 weeks or a single-agent treatment of physician’s choice (TPC), a control arm that allowed investigators to choose one of seven commonly used cytotoxic drugs (Perez et al. 2015). Median OS was numerically longer in the EP arm (12.4 vs. 10.3 months), but this difference was not statistically significant [hazard ratio (HR), 0.87; 95 % confidence interval (CI) 0.75–1.02; *P* = 0.084]. EP was associated with survival improvements (*P* < 0.05) relative to TPC in important, pre-defined subgroups with poor prognoses such as patients with liver metastases at baseline (median OS 10.9 vs. 8.3 months, respectively; HR 0.73) and those with stable, pre-treated brain metastasis at study entry (median OS 10.0 vs. 4.8 months, respectively; HR 0.51). Adverse event profiles differed between treatment arms, with a higher incidence of diarrhea reported among EP-treated patients and higher rates of neutropenia and neuropathy reported for TPC. We now report a more detailed analysis of the safety and tolerability of EP relative to TPC from the BEACON study.

## Methods

BEACON was a randomized, open-label, multicenter, phase 3 trial conducted in 11 countries (Perez et al. 2015). Briefly, eligible patients were adults with Eastern Cooperative Oncology Group (ECOG) performance status (PS) of 0 or 1 for whom single-agent chemotherapy for the treatment of locally recurrent or metastatic breast cancer was indicated. Eligible patients had received a minimum of two prior cytotoxic regimens for advanced disease and no more than five for breast cancer in any setting. Prior treatment with an anthracycline (unless contraindicated or not medically appropriate), a taxane and capecitabine was required. Patients with stable brain metastases were eligible, provided that local therapy was complete without ongoing need for corticosteroids.

Patients were randomized (1:1) to EP or a control arm (TPC) comprising one of seven commercially available chemotherapy drugs: eribulin, ixabepilone, vinorelbine, gemcitabine, paclitaxel, docetaxel or *nab*-paclitaxel. Etirinotecan pegol was administered at a dose of 145 mg/m^2^ every 21 days as a 90-min infusion. TPC was administered according to local practice, with the exceptions of eribulin and ixabepilone, which were administered in accordance with local product labeling.

All adverse events (AEs) were assessed with the National Cancer Institute (NCI) Common Terminology Criteria for Adverse Events (CTCAE) version 4.0. For TPC, dose delays, reductions, and discontinuations for AEs were made according to the prescribing information or local practice guidelines, and supportive care was administered at the investigator’s discretion. Dose modifications and specific supportive care regimens were defined in the protocol for EP. Prior to subsequent cycles, patients had to have adequate hematopoietic function; full resolution of diarrhea to grade 0 for at least 7 days without supportive antidiarrheal measures; and resolution of grade 3 electrolyte abnormalities to grade ≤1. Required dose reductions for specific toxicities are listed in Table 1; dose re-escalation was not allowed. Treatment could be delayed for up to 28 days to allow for recovery from toxicity. Patients who required treatment delays of >28 days were withdrawn from treatment, unless, in the investigator’s opinion and approved by the medical monitor, study continuation was deemed of benefit for the patient. Loperamide was dispensed to all patients randomized to EP at treatment start, with instruction to initiate therapy at first onset of diarrhea or loose stool and continue until resolution. Prophylactic anti-diarrheal medications were prohibited. Other supportive care measures were administered at the investigator’s discretion.

**Table 1:** Protocol-defined dose modifications for etirinotecan pegol in subsequent cycles based on worst toxicity in prior cycle

Adverse event by NCI CTCAE grade	Dose modification
Hematologic (thrombocytopenia, anemia, neutropenia, febrile neutropenia)	
Grade 1–2 (except ANC)	Maintain dose level
Grade 2 (ANC <1500/mm^3^)	First occurrence
	• Hold until ANC >1500/mm^3^
	• If screening ANC >2000/mm^3^; restart at reduced dose of 120 mg/m^2^
	• Do not dose reduce if screening ANC was ≥1500 but <2000/mm^3^
	Second occurrence
	• Decrease one dose level (to 90 or 120 mg/m^2^)
	Third occurrence
	• Discontinue treatment
Grade 3 (platelets <50 K or Hgb <8 g/dl)	First occurrence
	• Hold until resolved; restart at 120 mg/m^2^
	Second occurrence
	• Decrease additional dose level to 90 mg/m^2^
	Third occurrence
	• Discontinue treatment
Grade 4 (ANC <500/mm^3^ or platelets <25 K or Hgb <6.5 g/dl)	First occurrence
	• Hold until resolved (ANC >1500/mm^3^; Hgb >8 g/dl; platelets >50 K)
	• Restart at reduced dose of 120 mg/m^2^
	Second occurrence
	• Decrease additional dose level to 90 mg/m^2^
	Third occurrence
	• Discontinue treatment
Febrile neutropenia (grade 3: ANC <1000/mm^3^ with a single temperature >38.3 °C or a sustained temperature of ≥38 °C for more than 1 h)	First occurrence
	• Hold until resolved until grade 0–1 toxicity
	• Restart at reduced dose of 120 mg/m^2^
	Second occurrence
	• Decrease additional dose level to 90 mg/m^2^
	Third occurrence
	• Discontinue treatment
Diarrhea	
All grades	Confirm that diarrhea has been resolved for at least 7 days without use of supportive care prior to retreatment
Grade 1	Maintain dose level; consider prophylactic anti-diarrheal supportive care
Grade 2	First occurrence
	• Decrease one dose level to 120 mg/m^2^
	• Consider prophylactic anti-diarrheal supportive care
	Second occurrence
	• Decrease additional dose level to 90 mg/m^2^
	Third occurrence
	• Discontinue treatment
Grade 3–4	First occurrence:
	• Decrease two dose levels to 90 mg/m^2^
	• Use prophylactic anti-diarrheal supportive care
	Second occurrence
	• Provided that adequate supportive care was given previously, discontinue treatment
	• If the patient did not receive adequate prior supportive care, retreatment may be attempted if 2nd episode of grade 3 diarrhea
	• Discontinue treatment for 2nd episode of grade 4 diarrhea
Dehydration	
Grade 1	• Maintain dose level and consider appropriate prophylactic anti-emetic or anti-diarrheal supportive care
Grade 2	• Maintain dose level or decrease to 120 mg/m^2^ after first occurrence; consider appropriate prophylactic anti-emetic or anti-diarrheal supportive care
	• Decrease one dose level (to 120 or 90 mg/m^2^) after second occurrence; use appropriate prophylactic anti-emetic or anti-diarrheal supportive care
	• Up to two dose reductions are allowed
Grade 3–4	• Delay treatment until resolution to baseline or to grade 0
	• Decrease dose level to 90 mg/m^2^ after first occurrence; use appropriate prophylactic anti-emetic or anti-diarrheal supportive care
	• Discontinue after 2nd occurrence
Nausea/vomiting/abdominal pain	
Grade 1–2	• Maintain dose level and consider prophylactic anti-emetic supportive care
Grade 3	• Delay treatment until resolution to baseline or to grade 0
	• Decrease one dose level to 120 mg/m^2^ after first occurrence and use prophylactic anti-emetic supportive care
	• Decrease an additional dose level to 90 mg/m^2^ after second occurrence
	• Discontinue treatment after third occurrence
Grade 4	• Delay treatment until resolution to baseline or to grade 0
	• Decrease dose level to 90 mg/m^2^ after first occurrence and use prophylactic anti-emetic supportive care
	• Discontinue treatment after second occurrence

The frequencies of treatment-emergent AEs (TEAEs) and serious TEAEs (TESAEs) were tabulated by MedDRA preferred term and system organ class and by relationship to study drug. Summary statistics were prepared for the safety population, which comprises all patients who received at least one full or partial dose of assigned treatment. The treatment-emergent period was defined as the time between the first dose of study drug through 30 days after the last dose of study drug or the day prior to initiation of subsequent anti-cancer treatment, whichever occurred first. A TEAE was defined as an AE that was not present prior to treatment, but appeared following treatment or was present at treatment initiation and worsened during treatment. An AE that was present at treatment initiation, but resolved and then reappeared while the patient was on treatment, was also considered a TEAE, regardless of baseline intensity. A TESAE was defined as any untoward medical occurrence that, at any dose, resulted in death; was life-threatening; required or prolonged a hospitalization; resulted in persistent or significant disability or incapacity; was a congenital anomaly or birth defect; or was an important medical event that, based upon appropriate medical judgment, jeopardized the patient and required medical or surgical intervention to prevent one of the other outcomes that comprises an SAE. Odds ratio (OR) and exact confidence limits were calculated for AEs that occurred in at least 10 % of patients in the safety population. The time to onset of events was calculated from the first dose of the study treatment. Time to resolution was calculated from the onset date to the resolution date, death date, or date of start of new anti-cancer therapy. Because incidence rates are low in AEs of Special Interest, descriptive statistics were summarized based on patients who had the event or who had a resolution date when calculating time to onset and time to resolution of AEs of Special Interest.

The study was conducted according to the provisions of the Declaration of Helsinki and in accordance with International Conference on Harmonisation Good Clinical Practice standards, US Food and Drug Administration regulations, as well as any and all applicable federal, state and/or local laws and regulations. All patients gave written informed consent, and the study was approved by the relevant institutional review board or independent ethics committee at each site.

## Results

### Patients

The results of BEACON have been reported (Perez et al. 2015). Briefly, 852 patients were randomized and 831 comprise the safety population (425 treated with EP and 406 treated with TPC). Eribulin was the most frequently selected TPC agent (40 %), followed by vinorelbine (23 %) and gemcitabine (18 %). Baseline patient and disease characteristics were similar between treatment groups. The median age was 55 years in each group. Nearly all patients had received a prior anthracycline (96 %), and all had received a prior taxane and capecitabine. The median number of prior regimens for MBC was three in each group.

### Extent of exposure

The overall extent of exposure is summarized in Table 2. The median number of cycles was three for both treatment groups, and approximately 25 % of patients in each group received six or more cycles. The median duration of exposure, calculated between the date of first dose and last dose, to EP was 48.0 days (range 1–766 days) and 56.5 days for TPC (range 1–607 days). Median relative dose intensity, calculated as dose intensity divided by expected dose intensity, was higher in the EP arm (98.3 %) than in the TPC arm (92.8 %). The proportion of patients in each arm with any dose reduction, delay, or interruption due to AEs appeared similar. The number of cycles with dose reductions or delays due to AEs was lower with EP (11.8 %) than with TPC (19.7 %).

**Table 2:** Extent of exposure (safety population)

Endpoint	Etirinotecan pegol (n = 425)	TPC (n = 406)
Overall exposure duration, days		
Median	48.0	56.5
Mean (SD)	103.0 ± 117.8	99.5 ± 98.3
Cycles completed, n		
Median	3.0	3.0
Mean (SD)	5.5 ± 5.2	5.0 ± 4.2
Relative dose intensity, %^a^		
Median	98.3	92.8
Mean (SD)	92.6 ± 10.7	89.1 ± 16.2
Patient who had any dose reduction, n	117 (27.5 %)	115 (28.3 %)
Due to AE	117 (27.5 %)	108 (26.6 %)
Other reason(s)	0 (0.0 %)	9 (2.2 %)
Patients who had any dose delay, n	178 (41.9 %)	190 (46.8 %)
Due to AE	151 (35.5 %)	150 (36.9 %)
Other reason(s)	72 (16.9 %)	88 (21.7)
Patients who had any dose interruption, n	18 (4.2 %)	8. (2.0 %)
Due to AE	15 (3.5 %)	7 (1.7 %)
Other reason(s)	4 (0.9 %)	1 (0.2 %)
Number of cycles with dose reduction or delay due to AE^b^	276 (11.8 %)	397 (19.7 %)

*AE* adverse event, *TPC* treatment of physician’s choice

^a^Calculated as dose intensity divided by expected dose intensity; expected dose intensity (mg/m^2^ per week) equals the assigned dose (mg/m^2^) divided by planned cycle length (days) times 7

^b^Calculated as total number of dose reductions or delays due to AE divided by total of number of cycles received

### Adverse events

Almost all patients in both treatment arms experienced at least one TEAE, and most experienced at least one TEAE related to study drug (Table 3). The incidence of grade ≥3 TEAEs was significantly higher with TPC than EP (63.1 vs. 48.0 %, respectively; OR 0.54; 95 % CI 0.41–0.71; *P* < 0.0001). The most commonly reported grade ≥3 TEAEs in the EP arm were diarrhea and neutropenia (9.6 % for both). The most commonly reported grade ≥3 TEAE in the TPC arm was neutropenia (30.8 %). The odds of experiencing grade ≥3 neutropenia were significantly lower in the EP arm (OR 0.30; 95 % CI 0.21–0.42). The rates of all other grade ≥3 TEAEs were <5 % and generally similar between treatment arms.

**Table 3:** Summary of adverse events (safety population)

Endpoint	Etirinotecan pegol (n = 425)	TPC (n = 406)
Patients with at least 1 TEAE, n	417 (98.1 %)	405 (99.8 %)
Patients with at least 1 grade 3 or higher TEAE^a^, n	204 (48.0 %)	256 (63.1 %)
Patients with at least 1 TEAE related to study drug, n	394 (92.7 %)	356 (87.7 %)
Patients with at least 1 TEAE leading to study drug discontinuation	47 (11.1 %)	27 (6.7 %)
Patients with AE(s) leading to death^b^, n	5 (1.2 %)	8 (2.0 %)
Patients with at least 1 TESAE, n	128 (30.1 %)	129 (31.8 %)
Patients with at least 1 TESAE related to study drug, n	52 (12.2 %)	24 (5.9 %)

*TEAE* treatment emergent adverse event, *TESAE* treatment emergent serious adverse event, *TPC* treatment of physician’s choice

^a^ *P* < 0.0001

^b^Adverse event which is reported as the primary cause of death of the patient

Study drug discontinuation due to TEAEs was reported for 11.1 and 6.7 % of patients in the EP and TPC arms, respectively. There was a higher incidence of diarrhea leading to discontinuation in the EP arm (3.1 vs. 0 % with TPC) and a higher incidence of neuropathy leading to discontinuation in the TPC arm (2.2 vs. 0.2 % with EP). Although the rate of grade ≥3 neutropenia was lower in the EP arm, a greater proportion of patients were removed from EP treatment (2.8 %) for neutropenia compared to TPC (0.2 %).

A total of 128 (30.1 %) and 129 (31.8 %) patients in the EP and TPC arms experienced 225 and 206 TESAEs, respectively. Relatively few of these events were considered treatment-related (12.2 and 5.9 %, respectively). The most commonly reported TESAE in each group was pleural effusion (3.5 % for EP; 4.4 % for TPC). The most pronounced differences in TESAE rates were for diarrhea (4.0 % for EP vs. 0.5 % for TPC) and neutropenia (0.5 % for EP vs. 2.5 % for TPC).

### Deaths

A total of 327 patients (76.9 %) in the EP arm and 321 (79.1 %) in the TPC arm died during the study, primarily due to progressive disease [312 (73.4 %) and 304 (74.9 %), respectively]. Adverse events led to death in five patients (1.2 %) in the EP arm and eight (2.0 %) in the TPC arm. These events included one case each of pleural effusion, respiratory failure, myelodysplastic syndrome, pneumonia, and acute renal failure in the EP arm. Fatal events in the TPC arm included pleural effusion (n = 2) and one case each of respiratory failure, hepatic failure, fluid overload, lung infection, neutropenic sepsis, and septic shock.

### AEs of special interest

#### Diarrhea

Nearly two-thirds of EP-treated patients experienced diarrhea (Table 4). Most events were grade 1–2 in severity, and no patient in either treatment arm experienced grade 4 or 5 diarrhea. The median time to onset of grade ≥2 diarrhea in the EP arm was 39.5 days (range 1–471 days) versus 66.5 days (range 1–385 days) in the TPC arm. Median times to onset of grade 3 diarrhea were 43 days (range 3–488 days) and 7 days (range 1–79 days), respectively.

**Table 4:** Onset and resolution of diarrhea

	EP (n = 425)	TPC (n = 406)
Number of patients with diarrhea^a^, n (%)	281 (66.1)	80 (19.7)
Grade 1	177 (41.6)	52 (12.8)
Grade 2	63 (14.8)	23 (5.7)
Grade 3	41 (9.6)	5 (1.2)
Number of patients with diarrhea related to study drug^a^, n (%)	268 (63.1)	51 (12.6)
Grade 1	170 (40.0)	36 (8.9)
Grade 2	59 (13.9)	11 (2.7)
Grade 3	39 (9.2)	4 (1.0)
Onset of diarrhea grade 2 or higher, n (%)		
Number of patients	104	28
≥60 days	72 (16.9 %)	13 (3.2 %)
>60–90 days	10 (2.4 %)	8 (2.0 %)
>90–150 days	11 (2.6 %)	4 (1.0 %)
>150 days	11 (2.6 %)	3 (0.7 %)
Onset of diarrhea grade 3, n (%)		
Number of patients	41	5
≥60 days	25 (5.9 %)	4 (1.0 %)
>60–90 days	5 (1.2 %)	1 (0.2 %)
>90–150 days	5 (1.2 %)	0
>150 days	6 (1.4 %)	0

^a^Patients were counted once within each summary level. If a patient had more than one occurrence of the same event, the patient was counted once at the highest grade

The median duration of diarrhea of any grade was shorter in the EP arm (1.5 days; range 1–52 days) than in the TPC arm (3 days; range 1–123 days), while the median durations of grade 3 diarrhea were 6 days (range 1–31 days) and 4 days (range 1–21 days), respectively. In the EP arm, 47 patients (11.1 %) had a dose reduction and 63 patients (14.8 %) had a dose delay due to diarrhea. The corresponding proportions in the TPC treatment arm were 0.5 and 0.7 %, respectively. The median EP dose delay was 7 days (range 4–35 days). Of the 18 patients in the EP arm who permanently discontinued treatment due to diarrhea, 15 patients had resolution according to the investigator, with a median time to resolution of 28 days (range 12–81 days).

Overall, 60.4 % of patients in the EP arm and 12.1 % in the TPC arm received concomitant anti-diarrheal medications, primarily loperamide (58.3 and 6.4 % of patients in each respective arm). Additionally, six patients (1.4 %) in the EP arm were treated with octreotide, three (0.7 %) with tetracosactide, and one each (0.2 %) with lanreotide acetate and octreotide acetate.

#### Neutropenia

The incidence of grade ≥3 neutropenia was higher in the TPC arm than in the EP arm (Table 5). The median times to onset were 17 days (range 1–225 days) and 62 days (range 4–614 days), respectively. The median durations of neutropenia were 8 days (range 1–166 days) and 10 days (range 2–67 days), respectively. Similar proportions of patients experienced a dose reduction due to neutropenia (13.8 % on TPC and 14.4 % on EP), while more in the TPC arm experienced a dose delay (20.0 vs. 8.0 %, respectively). The median EP dose delay was 7 days (range 1–22 days). The median delay could not be calculated for the TPC arm because of regimen heterogeneity.

**Table 5:** Incidence of neutropenia

	EP (n = 425)	TPC (n = 406)
Number of patients with neutropenia, n (%)	111 (26.1)	174 (43.1)
Grade 1–2	70 (16.5)	50 (12.3)
Grade 3	32 (7.5)	79 (19.5)
Grade 4	9 (2.1)	45 (11.1)
Grade 5	0 (0)	1 (0.2)

A composite term encompassing neutropenia, febrile neutropenia, neutropenic sepsis, and neutrophil count decreased

Febrile neutropenia was relatively uncommon, occurring in three (0.7 %) and eight (2.0 %) patients in the EP and TPC groups, respectively. Pyrexia was observed slightly less frequently in the EP group (7.8 vs. 16.0 %).

Fourteen patients (3.3 %) and seven patients (1.7 %) in the EP and TPC arms, respectively, permanently discontinued treatment because of neutropenia. Of these, seven and six patients, respectively, had resolution according to the investigator, at a median time of 35 days (range 12–82 days) and 12 days (range 10–29 days), respectively.

More than twice as many patients in the TPC arm received colony stimulating factors (CSFs; 26.0 % for TPC vs. 11.9 % for EP). The most commonly prescribed CSFs were filgrastim (18.4 % and 8.9 %, respectively), pegfilgrastim (7.3 and 3.5 %, respectively), and lenograstim (1.4 and 0.5 %, respectively).

#### Neuropathy

The incidence of neuropathy was substantially higher in the TPC arm relative to EP (25.6 vs. 7.8 %, respectively), as was grade ≥3 neuropathy (3.7 vs. 0.5 %, respectively). Neuropathy was the most frequent cause for TPC drug discontinuation, occurring in 2.2 % of TPC-treated patients (vs. 0.2 % for EP).

### Subgroups of interest

#### Eribulin

The incidence of neutropenia, overall and grade ≥3, was higher among eribulin-treated patients than among EP-treated patients (Table 6) and time to onset was earlier (median times: 12.0 vs. 62.0 days, respectively). The median times to onset of grade ≥3 neutropenia were 16.0 and 120 days, respectively. Neuropathy, overall and grade ≥3, was also more commonly reported among eribulin-treated patients (overall: 32.3 vs. 7.8 %, respectively; grade ≥3: 4.3 vs. 0.5 %, respectively). The median duration of neuropathy was longer among eribulin-treated patients (22.0 vs. 10.5 days for EP) and more commonly associated with dose reductions (9.1 vs. 0.0 % for EP).

**Table 6:** Safety profiles in populations of interest

Adverse event, n (%)	Etirinotecan pegol (n = 425) (%)	Eribulin-treated patients (n = 164) (%)
Safety among eribulin-treated patients		
Neutropenia^a^, overall	26.1	39.0
Neutropenia^a^, grade 3 or higher	9.6	32.3
Neuropathy^b^, overall	7.8	32.3
Neuropathy^b^, grade 3 or higher	0.5	4.3

Grade 3 or higher TEAE, n (%)^c^	Etirinotecan pegol (n = 34)	TPC (n = 27)
Safety among patients with history of brain metastases		
At least one TEAE	17 (50.0)	19 (70.4)
Nausea	2 (5.9)	0
Pleural effusion	2 (5.9)	0
Syncope	2 (5.9)	0

Adverse event, n (%)	Etirinotecan pegol (n = 425) (%)	Eribulin-treated patients (n = 164) (%)
Safety among eribulin-treated patients		
Diarrhea	2 (5.9)	1 (3.7)
Neutropenia	5 (14.7)	9 (33.3)
Hyponatremia	0	2 (7.4)

*TEAE* treatment-emergent adverse event

^a^Composite term encompassing neutropenia, febrile neutropenia, neutropenic sepsis, and neutrophil count decreased

^b^Composite term encompassing acute polyneuropathy, critical illness polyneuropathy, mononeuropathy, mononeuropathy multiplex, multifocal motor neuropathy, neuronal neuropathy, neuropathy peripheral, peripheral motor neuropathy, peripheral sensorimotor neuropathy, peripheral sensory neuropathy, polyneuropathy, polyneuropathy idiopathic progressive, and polyneuropathy in malignant disease

^c^Adverse events reported in 5 % of more of patients in at least one treatment arm

#### Brain metastases

The safety profiles of EP and TPC among patients with a history of brain metastases were consistent with the overall safety population. More patients in the TPC arm experienced at least one grade ≥3 TEAE (70.4 vs. 50 % in the EP arm; Table 6). Grade ≥3 diarrhea occurred in 5.9 and 3.7 % of patients in the EP and TPC arms, respectively, and rates of grade ≥3 neutropenia were 14.7 and 33.3 %, respectively. Diarrhea was more common in the EP arm (55.9 vs. 18.5 % for TPC). Neuropathy-related events were more common in the TPC arm (25.6 vs. 7.8 % for EP).

## Discussion

This detailed comparison of the safety and tolerability of EP relative to TPC in the randomized phase 3 BEACON study demonstrates that EP is generally well tolerated, produces significantly fewer grade 3 and 4 toxicities, and does not share overlapping toxicities with commonly used MBC single-agent therapies, including eribulin. Fewer patients in the EP arm experienced a dose delay or dose reduction due to AEs, and median relative dose intensity was higher than in the TPC arm. The development of AEs can limit treatment exposure, jeopardize a patient’s likelihood of achieving maximal therapeutic benefit, and adversely affect QOL. The relatively favorable tolerability profile of EP in BEACON, coupled with statistically superior global health status and physical functioning in corresponding QOL analyses, is likely to translate into a clinically meaningful benefit for heavily pretreated patients with MBC (Cortes et al. 2015). In the context of the efficacy seen in the BEACON study, especially in certain patient subgroups, these data re-inforce the potential utility of EP in the treatment of women with MBC.

The safety profile of EP is consistent with expectations for a pegylated version of irinotecan. The clinical utility of conventional irinotecan is limited by suboptimal pharmacokinetics. When given intravenously at conventional doses, irinotecan produces relatively high peak plasma concentrations that are believed to be responsible for the cholinergic reactions, significant myelosuppression, and early-onset diarrhea seen in clinical practice (Kehrer et al. 2000; Masi et al. 2004; Takimoto et al. 2000; Herben et al. 1999). Additionally, the short terminal half-lives of the parent compound (9–14 h) and active metabolite (24–47 h) result in undetectable plasma levels of these cell-cycle specific agents within 1 week of administration (Kehrer et al. 2000; Chabot 1997; Pitot et al. 2000). A 21-day dosing interval therefore results in relatively short, intermittent tumor exposure to SN38 and peak plasma levels associated with toxicity. EP was specifically engineered to produce continuous exposure to SN38 without the high peak plasma levels (Hoch et al. 2014; Jameson et al. 2013). As expected, the incidence of neutropenia in BEACON was numerically lower in the EP arm than in the TPC arm, and neutropenic events appeared to be less severe. Approximately one-quarter of EP-treated patients developed neutropenia, and more than half of the cases were grade 1–2 in severity. The rates of grade 3–4 neutropenia were relatively low in the EP arm (9.6 %) compared to the TPC arm (30.6 %), and fewer patients in the EP arm required growth factor support. A greater proportion of EP-treated patients discontinued treatment due to neutropenia; there were, however, specific, protocol-mandated guidelines for managing neutropenia in the EP arm that likely contributed to differential discontinuation rates. Notably, the median time to onset of grade 3–4 neutropenia was substantially delayed with EP (120 vs. 16 days for TPC), demonstrating that patients were able to receive more courses of EP prior to the development of severe neutropenia relative to their counterparts in the TPC arm.

Diarrhea is a well-known side effect of irinotecan and, as expected, was relatively common among EP-treated patients in the BEACON trial. As with neutropenia, the time to onset of grade 3–4 diarrhea was longer with EP than with TPC. Indeed, this protracted time to diarrhea onset is consistent with the pharmacokinetic profile of EP, which eliminates the high peak plasma irinotecan concentrations associated with early-onset, cholinergic diarrhea. Strict protocol-mandated diarrhea management guidelines used in BEACON minimized the risk for grade ≥3 diarrhea. These guidelines were developed based on safety data from phase 2 trials. In a phase 2 trial initiated without guidelines, the incidence of grade 3 diarrhea was 23 % in patients with MBC who received EP 145 mg/m^2^ given every 3 weeks (Awada et al. 2013). In contrast, fewer than 10 % of EP-treated patients in BEACON experienced grade 3 diarrhea, and none experienced a grade 4 or grade 5 event.

Six of the seven agents comprising the TPC treatment options were microtubule inhibitors, including eribulin, for which neuropathy is the primary toxicity concern. As expected, the incidence of neuropathy, a cumbersome side effect that interferes significantly with QOL, was higher in the TPC arm, and neuropathy was the most common cause for TPC drug discontinuation. The lack of clinically significant neuropathy with EP therefore provides an attractive alternative for patients with MBC who have experienced or are at increased risk for neuropathy in view of prior therapies.

In summary, the BEACON study demonstrated that EP produces clinically meaningful outcomes (Perez et al. 2015) with a manageable toxicity profile, resulting in an overall favorable risk/benefit ratio for patients with heavily pretreated MBC. The lack of substantial overlapping toxicities with other agents used in this setting suggests that EP could be used sequentially with or in lieu of agents with different safety profiles. Importantly, the favorable safety profile was maintained among patients with a history of brain metastases who appeared to gain particular benefit from EP, providing further support for on-going clinical development in this patient population.
